# Neurologic Complications of Leprosy: A Case Series

**DOI:** 10.7759/cureus.59884

**Published:** 2024-05-08

**Authors:** Soukaina Benlamkadam, Klevor Raymond, Mohamed Chraa, Najib Kissani

**Affiliations:** 1 Neurology, Mohammed VI University Medical Center, Marrakesh, Marrakesh, MAR

**Keywords:** morocco, mycobacterium leprae, nerve conduction studies, peripheral neuropathy, skin, leprosy

## Abstract

Leprosy, caused by the *Mycobacterium leprae* complex, manifests as a chronic infection. Its hallmark presentation involves the neurocutaneous syndrome, characterized by peripheral nerve involvement and dermatologic lesions. Neurological complications significantly contribute to disability in leprosy patients. Peripheral neuropathy may manifest acutely or chronically, in either axonal or demyelinating forms, and can present as mononeuropathy, mononeuropathy multiplex, or polyneuropathy. The diverse clinical presentations emphasize the importance of considering leprosy in the differential diagnosis of peripheral neuropathy, enabling appropriate investigative approaches. Skin and nerve biopsies, slit skin smears, and nerve conduction studies serve as crucial diagnostic tools for identifying peripheral nerve involvement in leprosy. In this paper, we present three cases of leprosy with peripheral nerve involvement, discussing their clinical spectrum, diagnostic approach, and management.

## Introduction

Leprosy, a chronic infectious neurocutaneous disease, is caused by the alcohol and acid-resistant obligatory intracellular bacilli, namely *Mycobacterium leprae* and *M. lepromatosis*, collectively referred to as the *Mycobacterium leprae* complex [1]. It stands as one of the oldest known infections in humans, though its prevalence has significantly decreased in modern times, largely due to the concerted efforts of the World Health Organization (WHO). However, it remains predominantly endemic in certain regions worldwide [2]. The WHO's recent initiative, 'Toward Zero Leprosy', marks a determined stride toward the global elimination of this condition [3]. In Morocco, the milestone of eliminating leprosy as a public health concern was attained in 1991 [4].

Despite such achievements, the contagious nature of leprosy, along with its potential for functional and aesthetic complications, underscores its continued significance to healthcare providers. This is particularly relevant for neurologists, as leprosy should be considered in the differential diagnosis of certain patterns of peripheral nerve involvement. Thus, familiarity with the electroclinical neurologic phenotype of leprosy is imperative for neurologists. This would allow for early diagnosis and the timely instauration of a multidrug regimen as prescribed by the WHO [3]. 

In this paper, we present a series of three cases diagnosed with lepromatous peripheral neuropathy. Through a comprehensive review of the literature, we discuss the spectrum of presentations, diagnostic approaches, and available management options.

## Case presentation

Case 1

**Figure 1:**
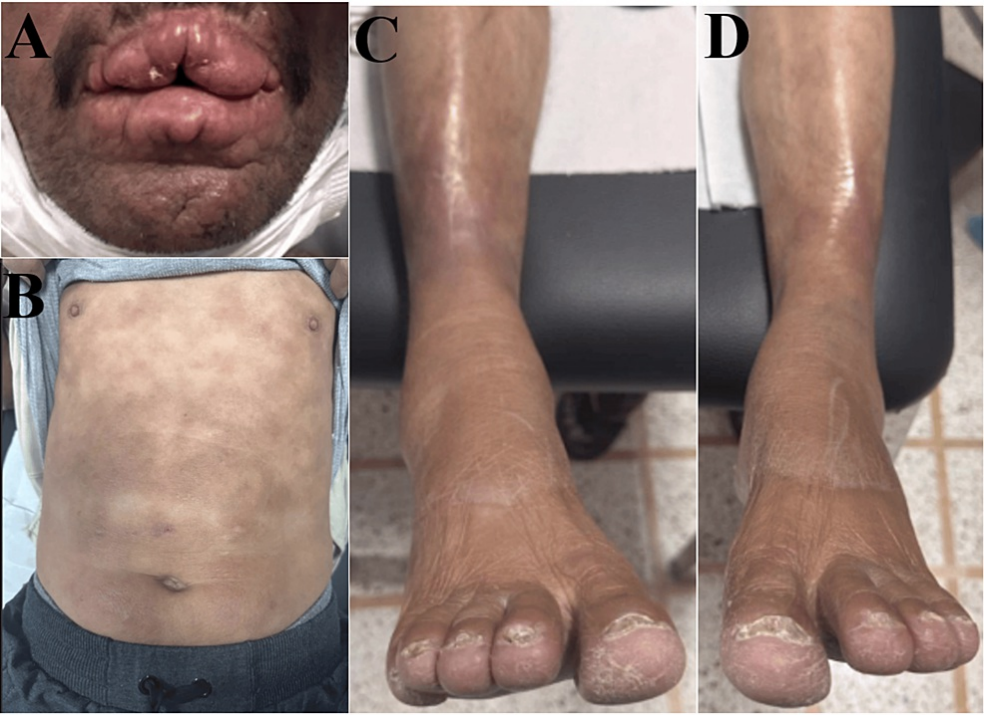
A: Macrochelia. B: Infiltrated erythematous/hyperpigmented lesions on torso. C and D: Shiny skin on shins with onychodystrophy and trachyonychia of toenails.

A 48-year-old male patient residing in a rural area presented with a three-year history of chronic dermatologic lesions and the progressive onset of reduced sensation in the lower limbs without pain or dysautonomic impairment. Upon examination, diffuse pruritic-infiltrated plaques were noted on the torso and all four limbs, accompanied by macrochelia (Figure 1). The sensation was reduced over the dermatologic lesions, and the patient also exhibited diminished temperature and pain sensation in both lower limbs. Force was preserved, and deep tendon reflexes were present. No limb deformities, or nerve thickening, or enlargement were observed, and the ophthalmologic examination was normal.

Nerve conduction studies (NCS) revealed axonal sensory-motor polyneuropathy (Tables 1, 2). A skin biopsy showed non-necrotizing granulomatous dermo-hypodermatitis with epithelioid and giant cells. Electrocardiogram (EKG) and cardiac sonography results were normal. 

**Table 1:** Motor nerve conduction studies (NCS) for case 1 NR: non-recordable, R: right, L: left. Normal values are indicated in brackets.

Motor Nerve Conduction Study			
Nerve	Latency	Amplitude	Velocity
R Ulnaris			
Wrist	3.2 (<4)	7.8 (>5.5)	-
Elbow	7.2	7.9	60.0 (>48)
L Ulnaris			
Wrist	4.1 (<4)	7.5 (>5.5)	-
Elbow	6.9	7.7	82.2 (>48)
R Medianus			
Wrist	3.9 (<4.2)	5.2 (>5)	-
Elbow	7.6	5.6	65.9 (>48)
L Medianus			
Wrist	3.1 (<4.2)	6.6 (>5)	-
Elbow	8.2	6.2	65.9 (>48)
R Peroneus			
Ankle	3.1 (<5.5)	3.4 (>3)	-
Fibula head	6.0	3.1	53.4 (>42)
L Peroneus			
Ankle	3.5 (<5.5)	3.9 (>3)	-
Fibula head	4.5	3.2	65.0 (>42)
R Tibial nerve	5.5 (<6.5)	3.7 (>3)	-
L Tibial nerve	5.8 (<6.5)	3.2 (>3)	-

**Table 2:** Sensory nerve conduction studies (NCS) for case 1 NR: non-recordable, R: right, L: left. Normal values are indicated in brackets.

Sensory Nerve Conduction Study			
Nerve	Latency	Amplitude	Velocity
R Ulnaris	1.8 (<4)	12.0 (>8)	89 (>45)
L Ulnaris	2.6 (<4)	12.0 (>8)	42.5 (>45)
R Medianus	1.8 (<4)	8.6 (>15)	80.6 (>45)
L Medianus	1.9 (<4)	6.3 (>15)	64.1 (>45)
R Suralis	4.1 (<4.5)	4.3 (>6)	45.6 (>40)
L Suralis	4.0 (<4.5)	2.5 (>6)	45.2 (>40)

Based on WHO criteria, a diagnosis of multibacillary leprosy with grade 2 impairment was established. The patient was initiated on multidrug therapy (MDT), which included Dapsone 100 mg daily, Clofazimine 300 mg monthly, Rifampin 600 mg monthly, and a daily dose of Clofazimine 50 mg for 12 months. To prevent leprosy reactions, MDT was supplemented with Prednisone 60 mg daily for four weeks, with a progressive tapering regimen thereafter. At three months of the treatment regimen, the patient reported improvement of sensation in the lower limbs and stable dermatologic lesions. 

Case 2

A 30-year-old patient, previously diagnosed with tuberculoid leprosy nine years ago, presented with a one-year history of progressively worsening sensory-motor impairment in all four limbs. The initial diagnosis of leprosy was made based on dermatologic manifestations and sensory-motor demyelinating multiple mononeuropathy. The patient acknowledged non-adherence to therapy upon initial diagnosis. Approximately one year ago, he reported progressive worsening of paresthesias and the appearance of new dermatologic lesions.

**Figure 2:**
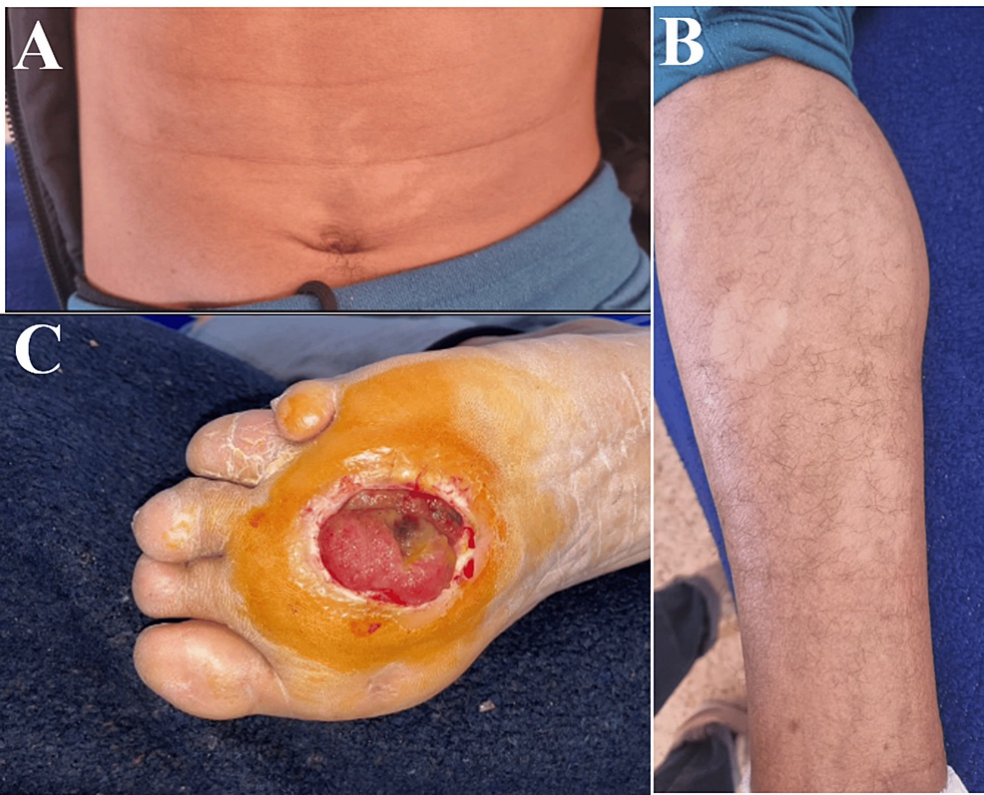
A and B: Achromic lesions on the abdomen and right leg. C: Malum perforans pedis of the left foot, with a clean depth, measuring 3 cm wide with non-inflamed, raised borders.

On examination, the patient exhibited distal weakness in the left upper (4/5 muscle strength) and lower limbs (4/5 muscle strength) with normal deep tendon reflexes. There was sensory impairment in the left upper limb and distally in both lower limbs in a sock distribution. Additionally, an ulcerating plantar lesion was observed at the central forefoot pressure point of the left foot (Figure 2).

Nerve conduction studies (NCS) revealed sensory-motor demyelinating multiple mononeuropathies (Tables 3, 4). A biopsy of the ulcerating lesion indicated signs of sub-acute and chronic ulcerations without specific or neoplastic findings.

**Table 3:** Motor nerve conduction studies (NCS) for case 2 NR: non-recordable, R: right, L: left. Normal values are indicated in brackets. *L Peroneus and L Tibial nerve not performed due to the proximity of electrodes to foot ulceration.

Motor Nerve Conduction Study			
Nerve	Latency	Amplitude	Velocity
R Ulnaris			
Wrist	4.2 (<4)	8.2 (>5.5)	-
Elbow	9.3	8.0	49.2 (>48)
L Ulnaris	NR	NR	NR
R Medianus			
Wrist	5.2 (<4.2)	10.2 (>5)	-
Elbow	10.2	10.8	47.2 (>48)
L Medianus			
Wrist	5.7 (<4.2)	1.7 (>5)	-
Elbow	11.0	4.2	45.5 (>48)
R Peroneus			
Ankle	4.2 (<6.5)	5.9 (>3)	-
Fibula head	13.4	4.0	37.7 (>42)
L Peroneus*	-	-	-
R Tibial nerve	5.6 (<6.5)	1.3 (>3)	-
L Tibial nerve*	-	-	-

**Table 4:** Sensory nerve conduction studies (NCS) for case 2 NR: non-recordable, R: right, L: left. Normal values are indicated in brackets. *L Suralis not performed due to the proximity of electrodes to foot ulceration.

Sensory Nerve Conduction Study			
Nerve	Latency	Amplitude	Velocity
R Ulnaris	1.7 (<4)	15.4 (>8)	73.3 (>45)
L Ulnaris	1.7 (<4)	19.3 (>8)	86.2 (>45)
R Medianus	3.3 (<4)	3.5 (>15)	43.7 (>45)
L Medianus	1.7 (<4)	15.4 (>15)	73.3 (>45)
R Suralis	3.1 (<4.5)	12 (>6)	56.6 (>40)
L Suralis*	-	-	-

A diagnosis of leprosy relapse was established, classified as a paucibacillary form with grade 2 impairment according to WHO criteria. The patient was initiated on multidrug therapy (MDT), which included Dapsone 100 mg daily, Clofazimine 300 mg monthly, and Rifampin 600 mg monthly, along with a daily dose of Clofazimine 50 mg for six months. Prednisone 60 mg daily for four weeks, with a progressive tapering regimen, was administered to prevent leprosy reactions. Gabapentin (1200 mg) was prescribed for paresthesias. After three months of treatment, the patient no longer reported paresthesias; however, he reported no changes in muscle strength in the left upper and lower limbs. He was referred to dermatology for the management of the foot ulcer. 

Case 3

**Figure 3:**
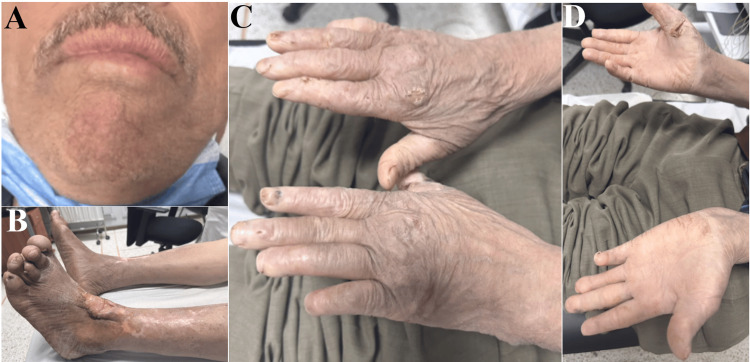
A: Erythematous papules of the chin. B: Toe deformity and an ulcerating lesion over the anterior ankle with hypertrophic granulation tissue. C and D: Dry skin with erosive and hyperkeratotic lesions of hands, thickened digits, and onychodystrophy.

A 62-year-old male patient, presenting with a two-month history of dermatologic lesions on the face, was referred to neurology for evaluation due to insensitivity to temperature in distal limbs persisting for two years. Upon examination, the patient exhibited infiltrated erythematous lesions on the face and ear lobes, along with achromic lesions on the trunk. Additionally, an ulceration was noted on the left leg following a burn accident. Deformities of the fingers and toes were also observed (Figure 3). Reduced temperature and pain sensation were noted in a glove and stocking distribution. The ophthalmologic examination yielded normal results.

Nerve conduction studies (NCS) revealed left tibial axonal mononeuropathy with probable small-fiber neuropathy in all four limbs (Table 5). Skin slit smear samples collected from the eyebrow arches, ear lobes, and nasal mucosa revealed positive acid-fast bacilli with a bacillary index of 4+. X-ray imaging of the distal limbs showed evidence of acro-osteolysis and interarticular narrowing.

**Table 5:** Motor nerve conduction studies (NCS) for case 3 NR: non-recordable, R: right, L: left. Normal values are indicated in brackets.

Motor Nerve Conduction Study			
Nerve	Latency	Amplitude	Velocity
R Ulnaris			
Wrist	2.7 (<4)	10.1 (>5.5)	-
Elbow	7.7	9.5	50.4 (>48)
L Ulnaris			
Wrist	2.6 (<4)	9 (>5.5)	-
Elbow	7.4	7.9	50.4 (>48)
R Medianus			
Wrist	3.3 (<4.2)	7.7 (>5)	-
Elbow	7.9	6.4	51.2 (>48)
L Medianus			
Wrist	3.3 (<4.2)	6.6 (>5)	-
Elbow	8.3	6.2	65.9 (>48)
R Peroneus			
Ankle	3.9 (<5.5)	3.7 (>3)	-
Fibula head	10.6	3.1	45.5 (>42)
L Peroneus			
Ankle	3.3 (<5.5)	3.7 (>3)	-
Fibula head	9.5	3.0	45.5 (>42)
R Tibial nerve	5.6 (<6.5)	6.7 (>3)	-
L Tibial nerve	6.3(<6.5)	3.3 (>3)	-

Based on WHO criteria, a diagnosis of multibacillary leprosy with grade 2 impairment was established. The patient was initiated on multidrug therapy (MDT), which included Dapsone 100 mg daily, Clofazimine 300 mg monthly, and Rifampin 600 mg monthly, along with a daily dose of Clofazimine 50 mg for 12 months. Prednisone 60 mg daily for four weeks, with a progressive tapering regimen, was administered to prevent leprosy reactions. At three months into the treatment regimen, the patient reported persistent sensory impairment without any worsening since starting treatment. Dermatologic lesions remained stable without the appearance of any new lesions.

## Discussion

Although Morocco has met the WHO criteria for eliminating leprosy as a public health problem, there are still annual reports of new cases. In 2022, the country reported 14 new cases [5]. There is a pressing need for improved case detection, with neurologists playing a pivotal role in both the diagnosis and management of leprosy. A thorough neurologic examination, along with nerve conduction studies and a nerve biopsy, are crucial steps in the neurological diagnostic process.

Peripheral nerve involvement stands as the primary neurological manifestation of leprosy, attributed to the predilection of *M. leprae *for Schwann cells [6]. This results in demyelination. Axonal degeneration can also be encountered in leprosy and peripheral neuropathy. Peripheral nerve involvement can stem from the direct destruction of cells by the mycobacteria or the host's immunologic response. Host immune responses may lead to the formation of granulomas or abscesses, causing nerve thickening or neuritis. Granulomas can induce focal nerve impairment, leading to mononeuropathy. The accumulation of granulomas in multiple nerves may progress to mononeuropathy multiplex and eventually manifest as a clinical picture resembling polyneuropathy. However, leprosy reactions, which are immunologic flare-ups, can result in either mononeuritis multiplex or polyneuropathy [7,8]. Even after treatment, the presence of bacterial remnants in the nerves could trigger immune events, leading to neuropathy. Peripheral neuropathy in leprosy can manifest as acute, subacute, or chronic [9].

In the neurology clinic, a variety of clinical scenarios may prompt the diagnosis of leprosy. The most apparent scenario involves patients presenting with hypoesthetic dermatologic lesions. Typically, these patients are initially assessed by general practitioners or dermatologists. Since examining sensory function over a skin lesion is not standard practice, consideration of leprosy is necessary to test for reduced sensory function over the lesion. Occasionally, patients with dermatologic lesions may report sensory or motor dysfunction, prompting a referral to neurology. However, leprosy can also manifest solely as pure neuritic leprosy without dermatologic manifestations [9]. In such cases, the onus is on the neurologist to include leprosy in the list of possible diagnoses.

The cases presented in this series encompass a wide spectrum of leprosy neuropathy presentations, ranging from acute to chronic, with various distribution patterns including mononeuropathy, mononeuropathy multiplex, and polyneuropathy, as well as underlying axonal and demyelinating pathologies. Two cases were attributed to *de novo* infections, while one was due to a relapse. In one case, small fiber involvement was suspected, warranting consideration of a skin biopsy. However, it was deemed unnecessary, as the diagnosis of leprosy neuropathy was evident from other investigations.

In addition to nerve conduction studies and a biopsy of the skin and nerves, other investigative tools for leprosy neuropathy include nerve magnetic resonance imaging and nerve ultrasonography. However, these diagnostic tools are seldom utilized in routine practice and require specialized expertise. Additional investigative modalities may be warranted, particularly in cases of pure neuritic leprosy presentation. These may include spinal taps, immunoelectrophoresis of serum proteins and spinal fluid, C-reactive protein, and erythrocyte sedimentation rate, among others. These tests primarily serve to rule out differential diagnoses. For instance, in cases of mononeuritis multiplex, differential diagnoses to consider include vasculitis, sarcoidosis, hereditary neuropathy with liability to pressure palsies (HNPP), and infiltration with cancer or lymphoma. In cases of nerve thickening, conditions such as chronic inflammatory demyelinating polyneuropathy, amyloid neuropathy, neoplastic infiltration with tumors, neurofibromatosis, Charcot Marie Tooth type 1A, HNPP, and Refsum’s disease should be considered [10,11].

The management of neuropathies in leprosy involves two main components: medication and physiotherapy. It is crucial not to delay treatment for leprosy neuropathy, as this can lead to irreversible nerve damage and disability. Multidrug therapy is the cornerstone of managing neuropathy in leprosy, with the WHO-recommended standard regimen being implemented for all patients. Clofazimine, despite being an antibiotic, possesses anti-inflammatory effects and may be beneficial in managing leprosy reactions [12].

In our center, steroid therapy is initiated concurrently with antibiotics to prevent leprosy reactions. Although this practice may not be standard in other centers and lacks consensus, it helps mitigate the risk of this potentially severe complication and encourages patients' adherence to the long-term treatment plan. Steroids are also effective in treating sensory symptoms such as paresthesias, dysesthesias, or neuropathic pain. However, antidepressants and antiepileptics are particularly noteworthy in managing these symptoms [13].

Surgery is another option for managing leprosy neuropathy, but it is reserved for select cases. The most common procedures involve repairing disfigurements and decompressing nerves [14].

Upon treatment completion, a 5- to 10-year follow-up period is recommended. This allows for the detection of relapse or immune reactions. Relapse is very low in well-treated patients. In the absence of treatment, patients worsen with aesthetic complications such as facial disfigurement or permanent nerve damage. Patients could also present leprosy reactions, which could be fatal. Prompt treatment prevents the progression of tissue damage, which sometimes remains irreversible without adjunct treatment like surgical reparation. Skin lesions typically disappear within the first year of treatment [10].

## Conclusions

Leprosy typically presents as a neurocutaneous syndrome, impacting both the functionality and aesthetics of infected individuals. Neurological manifestations are common and can manifest in various forms, even in treated patients, emphasizing the crucial role of neurologists in recognizing, diagnosing, and managing leprosy. It is particularly important to consider the possibility of leprosy neuropathy in patients presenting with neuropathy alongside dermatologic manifestations. However, leprosy can also manifest solely as isolated peripheral neuropathy, which should be considered, especially in endemic regions and when diagnostic workup yields inconclusive results. Management necessitates a well-defined treatment regimen, incorporating physiotherapy, and efforts to combat stigma.
